# Augmentation of 5-lipoxygenase activity and expression during dengue serotype-2 infection

**DOI:** 10.1186/1743-422X-10-322

**Published:** 2013-10-30

**Authors:** Wai Mun Loke, Angelia Yee Chow, Karen Lam Mok Sing, Chung-Yung J Lee, Barry Halliwell, Erle CH Lim, Amy ML Quek, Eng Eong Ooi, Raymond CS Seet

**Affiliations:** 1 Department of Medicine, Yong Loo Lin School of Medicine, National University of Singapore, 1E, NUHS Tower Block, Kent Ridge Road, Singapore; 2 Program in Emerging Infectious Diseases, Duke-NUS Graduate Medical School, Singapore; 3 Department of Biochemistry, Yong Loo Lin School of Medicine, National University of Singapore, Singapore

**Keywords:** Inflammation, Leukotriene B_4_, Dengue, 5-lipoxygenase activities

## Abstract

**Background:**

Leukotriene B_4_, a 5-lipoxygenase product of arachidonic acid with potent chemotactic effects on neutrophils, has not been assessed in dengue patients. In this study, plasma leukotriene B_4_ and serum high-sensitivity C-reactive protein levels were determined in adult patients during the febrile, convalescent and defervescent stages of dengue serotype-2 (DENV-2) infection, and compared with those of age--matched healthy and non-dengue febrile subjects. *In vitro* studies were performed to examine the effects of live and heat-inactivated DENV-2 on the activities and expression of 5-lipoxygenase in human neutrophils.

**Results:**

Plasma leukotriene B_4_ was elevated during the febrile stages of dengue infection compared to levels during convalescence and in study controls. Plasma leukotriene B_4_ also correlated with serum high-sensitivity C-reactive protein in dengue patients (febrile, r = 0.91, *p* < 0.001; defervescence, r = 0.87, *p* < 0.001; convalescence, r = 0.87, *p* < 0.001). Exposure of human neutrophils to DENV-2 resulted in a significant rise in leukotriene B_4_; the extent of increase, however, did not differ between exposure to live and heat-inactivated DENV-2. Pre-incubation of either live or heat-inactivated DENV-2 resulted in reduced leukotriene B_4_ release by neutrophils, indicating that contact with dengue antigens (and not replication) triggers the neutrophil response. Production of leukotriene B_4_ was associated with an increase in 5-lipoxygenase expression in human neutrophils; addition of MK886 (a 5-lipoxygenase activating protein inhibitor) attenuated further increase in leukotriene B_4_ production.

**Conclusion:**

These findings provide important clinical and mechanistic data on the involvement of 5-lipoxygenase and its metabolites in dengue infection. Further studies are needed to elucidate the therapeutic implications of these findings.

## Background

Dengue is an important infectious cause of morbidity in tropical and subtropical countries. It is estimated that 250,000 cases of severe dengue infections and at least 100 million cases of dengue fever occur annually [1,2]. Following an initial infection by dengue virus that is transmitted primarily by the *Aedes aegypti* mosquito, high fever develops in the first 2–7 days (febrile stage) where viral replication takes place [1]. Thereafter, fever ceases (defervescent stage) and within 2 days, viremia falls to undetectable levels and clinical recovery to health begins (convalescent stage) [1,3]. Rapid onset of inflammation and activation of effective cellular immune responses limit the spread of virus during the initial stages of infection [1,3]. Among the effector cells playing a role in the inflammatory and immune responses are the polymorphonuclear leukocytes [4,5].

Neutrophils, the most abundant circulating polymorphonuclear leukocytes, are the first cell type to migrate from blood vessels before reaching infectious loci within the peripheral tissues. Neutrophils play a pivotal role in the phagocytosis of particles, production of reactive oxygen species (O_2_, HOCl and H_2_O_2_), lipid mediators (such as leukotrienes, prostaglandins, lipoxins and platelet-activating factors) and secretion of anti-microbial proteins [6,7]. One such well-characterized proinflammatory lipid mediator produced by neutrophils is leukotriene B_4_ (LTB_4_), a tetraunsaturated 20-carbon chain fatty acid derived by the enzymatic actions of 5-lipoxygenase and leukotriene A_4_ hydrolases on arachidonic acid [8]. LTB_4_ is involved in several inflammatory diseases (such as myocardial infarction and stroke) [9] and is capable of inducing antimicrobial responses [10]. By modulating cell surface G protein-coupled receptors (BLT1 and BLT2) [11], LTB_4_ promotes migration of neutrophils, monocytes and macrophages to sites of inflammation [12]. The level of C-reactive protein (CRP) is higher in diseases in which inflammation is implicated [3,13,14]. Greater levels of CRP have been observed among dengue patients compared with age-matched non-dengue controls [3,15] and among those with greater dengue severity [15].

To assess the relevance of the 5-lipoxygenase pathway in the pathogenesis of dengue infection, plasma LTB_4_ and serum high-sensitivity C-reactive protein (hsCRP) were measured in a cohort of patients with acute dengue virus serotype-2 (DENV-2) infection and age-matched controls. To determine their temporal changes, these markers were assessed during the different clinical stages of dengue infection (febrile, defervescence and convalescence). *In vitro* studies were performed to examine the effects of live and heat-inactivated DENV-2 on activities and expression of 5-lipoxygenase in human neutrophils.

## Results

Plasma LTB_4_ was significantly elevated during the febrile and defervescent stages of dengue infection compared with healthy and non-dengue febrile controls (*p* < 0.05, Table 1). These levels decreased further, approaching those of healthy controls and during convalescence (*p* < 0.001, Table 1). Serum hsCRP concentrations were higher in patients with DENV-2, especially during the febrile stage of dengue illness (Table 1). No significant difference was observed during disease convalescence and healthy controls for both plasma LTB_4_ and serum hsCRP (Table 1). Plasma LTB_4_ and serum hsCRP in non-dengue febrile controls were also significantly higher compared with healthy controls (*p* < 0.05, Table 1). Plasma arachidonic acid (AA) and PLA_2_ activities were not altered throughout the different stages of DENV-2 infection (Table 1).

**Table 1:** Circulating concentrations (mean ± SD) of measured parameters in patients with dengue serotype-2 virus infection during the febrile stage, defervescence and convalescence (N = 15), in healthy controls (N = 20) and in non-dengue febrile controls (N = 15)

**Parameters**	**Febrile**	**Defervescence**	**Convalescence**	**Healthy controls**	**Non-dengue febrile controls**
Plasma LTB_4_ (*p*mol/μmol AA)	0.35 (0.35)^b^	0.38 (0.34)^b^	0.01 (0.02)^c^	0.01 (0.01)^c^	0.17 (0.12)^d^
Serum hsCRP (mg/L)	6.10 (6.70)^b^	3.30 (3.00)^b^	0.60 (1.50)^c^	1.02 (1.01)^c^	3.36 (3.69)^b^
Plasma arachidonic acid (μg/mL)	89.0 (26.4)^a^	95.8 (24.3)^a^	110.0 (20.2)^a^	86.5 (19.7)^a^	87.7 (22.5)^a^
Plasma phospholipase A_2_ activity (nmol/ min/mL)	4.3 (0.9)^a^	4.4 (1.0)^a^	4.2 (0.9)^a^	4.2 (1.1)^a^	-

Data are summarized as mean (standard deviation).

^a^Normally distributed data with the same subscripts are not significantly different with each other using one-way ANOVA analyses with Bonferroni adjustment.

^b,c,d^Non-parametric data with the same subscripts are not significantly different with each other using Kruskal-Wallis analysis with Dunn’s adjustment.

Plasma LTB_4_ correlated significantly with serum hsCRP in patients with DENV-2 during the different stages of infection (febrile, r = 0.91, *p* < 0.001; defervescent, r = 0.87, *p* < 0.001; convalescent, r = 0.87, *p* < 0.001). A reduction in plasma LTB_4_ between the febrile and convalescent stages of illness also correlated with the changes in serum hsCRP concentration (r = 0.86, *p* < 0.001). These associations, however, were not observed in non-dengue febrile controls.

**Figure 1:**
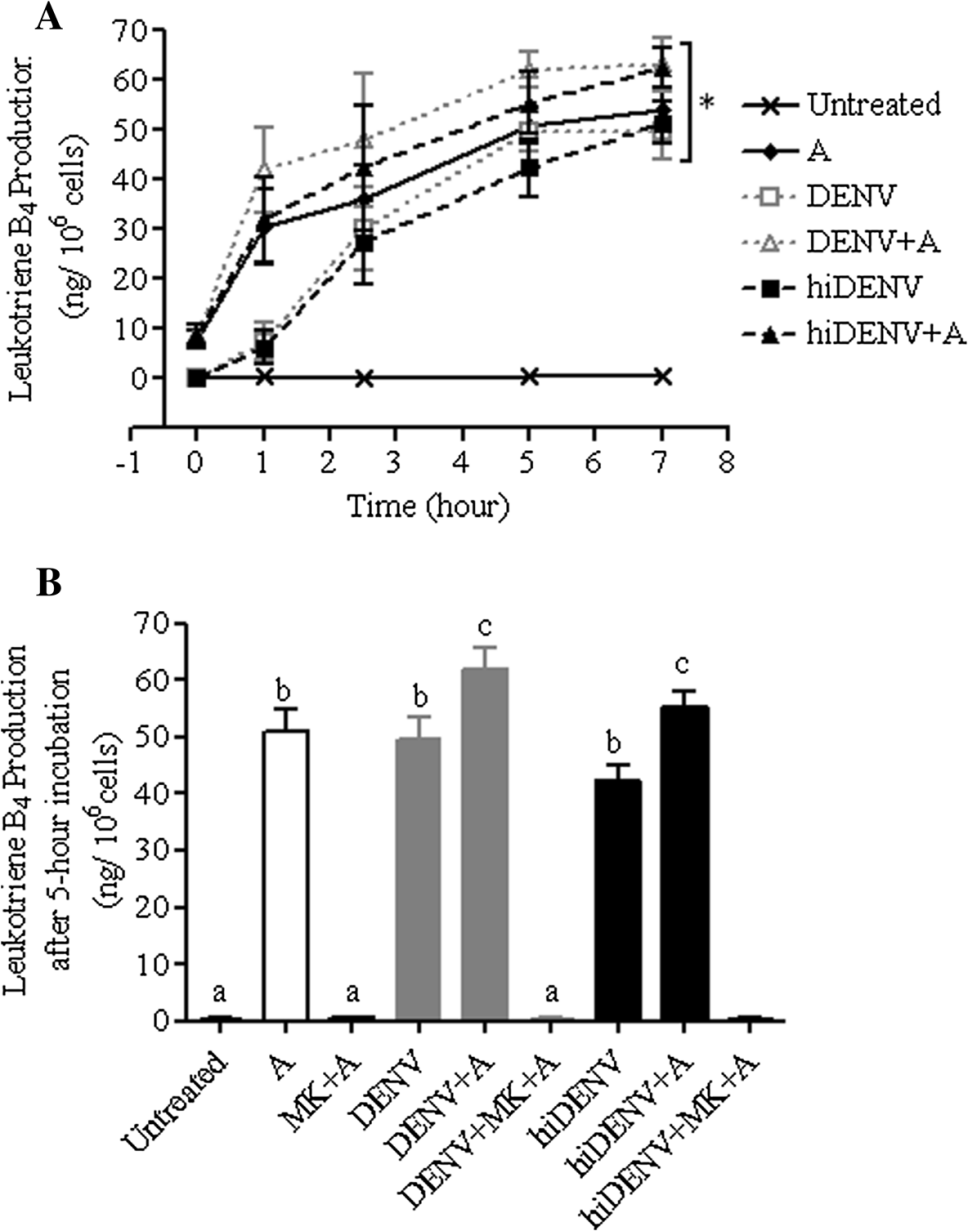
**Production of leukotriene B**_**4 **_**by neutrophils incubated with or without DENV-2. (A)** Production of leukotriene B_4_ (ng/10^6^ cells) by freshly isolated peripheral neutrophils over 7-hour incubation in the presence and absence of live dengue viruses exposure (DENV-2), heat-inactivated dengue viruses exposure (hiDENV-2), and A23187 **(A)** treatment (N = 5). * *p* < 0.05 vs untreated cells using one-way ANOVA analysis of the area under the curves with Bonferroni adjustments. **(B)** Leukotriene B_4_ production (ng/10^6^ cells) from freshly isolated peripheral neutrophils after 5 hours of incubation with or without live dengue viruses exposure (DENV-2), heat-inactivated dengue viruses exposure (hiDENV-2), MK886 (MK) and A23187 **(A)** treatment (N = 5). Bars with the same subscripts are not significantly different from each other using one-way ANOVA analysis with Bonferroni adjustments.

The effects of exposure to live and heat-inactivated DENV-2 on the 5-lipoxygenase pathway were examined using freshly isolated peripheral neutrophils. These neutrophils were incubated with or without DENV-2 at a multiplicity of infection of 1 over a period of 7 hours before these were stimulated with calcium ionophore A23187. In the absence of exogenous AA, no difference in LTB_4_ production in neutrophils was observed. Exogenous AA was added to the incubation medium to eliminate the potential confounding effects of phospholipase A_2_ (PLA_2_) and to examine the consequences of DENV-2 on 5-lipoxygenase in human neutrophils. Figure 1A shows production of LTB_4_ by freshly isolated neutrophils treated with DENV-2 during an incubation period of 7 hours. Release of LTB_4_ from human neutrophils exposed to DENV-2 was maximal five hours following incubation, before reaching a plateau. By contrast, neutrophils that were not exposed to DENV-2 did not produce detectable amounts of LTB_4_.

As expected, production of LTB_4_ from human neutrophils was suppressed by MK886 (a 5-lipoxygenase activating protein inhibitor) (Figure 1B). Neutrophils incubated with DENV-2 alone produced comparable amounts of LTB_4_ as those stimulated with A23187. There were no differences in production of LTB_4_ by neutrophils treated with live or heat-inactivated DENV-2 (Figures 1A and 1B), suggesting that DENV-2 infectivity is not a pre-requisite for LTB_4_ production. Neutrophils treated with both DENV-2 and A23187 produced significantly higher amounts of LTB_4_ than those treated with either the virus or A23187 (*p* < 0.05 vs A23187 stimulated neutrophils, Figure 1B). Addition of MK886 attenuated the production of LTB_4_ in the presence of DENV-2.

**Figure 2:**
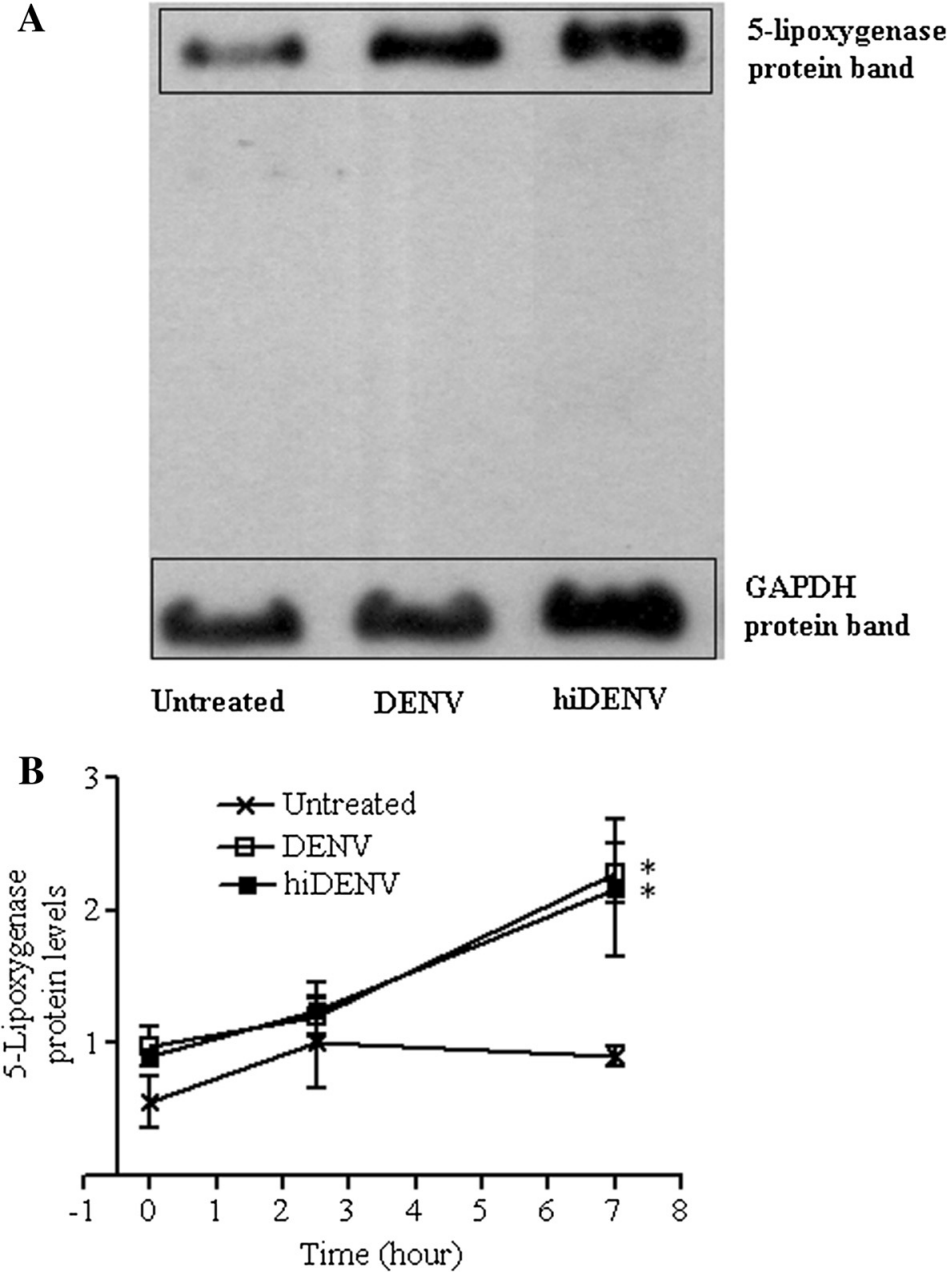
**5-Lipoxygenase expression by neutrophils incubated with or without dengue viruses.** The proteins were isolated from neutrophils and were electrophoretically transferred from the gel onto Hybond-C Extranitrocellulose membrane. The membrane was then cut, based on the size of proteins of interest, to separate the 5-lipoxygenase (79 kDa) and the control protein GAPDH (39 kDa). **(A)** Representative films from 5-lipoxygenase and GAPDH Western blots performed with untreated neutrophils and neutrophils incubated with live (DENV-2) or heat-inactivated dengue viruses (hiDENV-2). **(B)** Expression of 5-lipoxygenase protein by untreated neutrophils (×) and neutrophils exposed to live (DENV-2, □) or heat-inactivated dengue viruses (hiDENV-2, ■) (N = 3). * *p* < 0.05 vs untreated cells using one-way ANOVA analysis of the area under the curves with Bonferroni adjustments.

**Figure 3:**
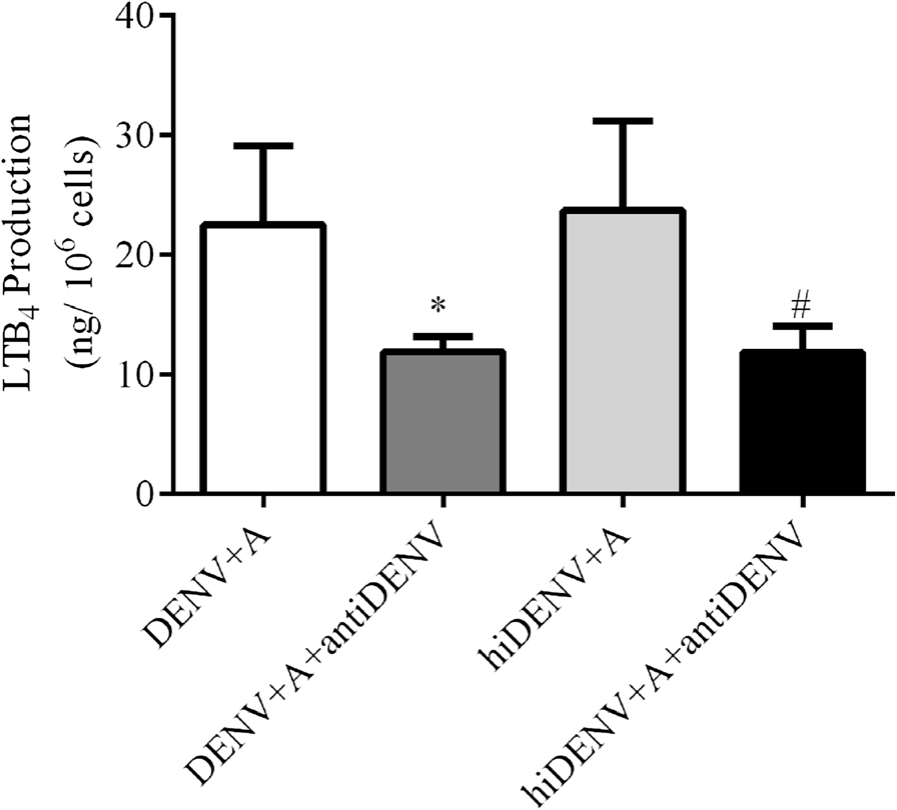
**Production of leukotriene B**_**4 **_**by neutrophils incubated with or without anti-DENV-2 antibody.** Production of leukotriene B_4_ (ng/10^6^ cells) from freshly isolated peripheral neutrophils after 5-hour incubation in the presence and absence of anti-DENV-2 antibody (antiDENV), with live DENV-2 (DENV), heat-inactivated DENV-2 (hiDENV) and A23187 **(A)** treatment (N = 5). * *p* < 0.05 vs DENV + A using one-way ANOVA analysis with Bonferroni adjustments. # *p* < 0.05 vs hiDENV + A using one-way ANOVA analysis with Bonferroni adjustments.

Representative 5-lipoxygenase and glyceraldehyde-3-phosphate dehydrogenase (GAPDH) Western blots performed with neutrophils incubated with or without DENV-2 are shown in Figure 2A. The levels of 5-lipoxygenase protein in neutrophils were significantly higher after incubation with DENV-2 (*p* < 0.05 vs untreated cells, Figure 2B). Introduction of MK886 did not influence the expression of 5-lipoxygenase protein in neutrophils exposed to DENV-2 (data not shown). Following incubation of neutrophils with DENV-2, the amount of LTB_4_ produced correlated significantly with the expression of 5-lipoxygenase protein in neutrophils at 0 and 7 hour time-points (r = 0.54, *p* < 0.05 at time 0 hour; r = 0.65, *p* < 0.05 at time 7 hour), but not at 2.5 hour. Despite prolonged incubation for 72-hours of DENV-2 in neutrophil suspension, immunofluorescence assays did not detect the presence of DENV-2 in neutrophils, indicating the inability of DENV-2 to infect neutrophils *in vitro* (data not shown). Furthermore, addition of anti-DENV-2 antibody to neutrophils inhibited LTB_4_ production compared to neutrophils exposed to live DENV-2 and hiDENV-2 without anti-dengue antibody (Figure 3). These findings collectively suggest that exposure to DENV but not viral replication in neutrophil triggers LTB_4_ production.

## Discussion

Previous studies have implicated 5-lipoxygenase and LTB_4_ in the pathogenesis of various microbial diseases [16,17]. An interaction between the Epstein-Barr virus and monocytes is thought to upregulate LTB_4_ formation by enhancing 5-lipoxygenase and phospholipase activities [18]. Another study observed greater expressions of 5-lipoxygenase and LTB_4_ in vascular smooth muscle cells exposed to the human cytomegalovirus virus [19]. Addition of human immunodeficiency virus-1 envelope proteins stimulates release of arachidonic acid-derived 5-lipoxygenase products (such as LTB_4_ and LTC_4_), from human mononuclear cells via signalling through the CD4 molecules (T-helper immune cells) [20]. In pneumonia patients, an increase in LTC_4_ in the respiratory tract secretion is associated with the presence of respiratory syncytial virus [21]. A detailed understanding on the roles of lipoxygenases in dengue infection is necessary as emerging data are beginning to suggest that leukotrienes produced during an infection may exert antimicrobial activities. In healthy subjects, nasal administration of LTB_4_ increases nasal neutrophil recruitment and activity [22] while, *in vitro*, HIV viral replication is significantly reduced after LTB_4_ administration [23].

In the current study, DENV-2 infection was shown to increase the levels of circulatory LTB_4_ as compared with age-matched controls, and the rise was apparent even when comparisons were made with patients with febrile illnesses that were not caused by dengue infection. Following exposure to DENV-2 *in vitro*, a time-dependant release of LTB_4_ was observed before reaching a plateau approximately five hours following DENV-2 exposure. The correlation between LTB_4_ production and 5-lipoxygenase expression is biologically important as this suggests that the observed rise in circulatory LTB_4_ may (in part) be explained by the release of LTB_4_ from neutrophils. Despite administration of live and heat-inactivated DENV-2, we were not able to demonstrate differences in the production of LTB_4_ and expression of 5-lipoxygenase in neutrophils. Nor was there evidence to indicate the ability of DENV-2 to infect neutrophils and trigger circulatory release of LTB_4_. Furthermore, the addition of anti-dengue antibody reduced LTB_4_ production by neutrophils stimulated by either live or hi-DENV-2. Collectively, these findings suggest that viral infectivity and replication is not a requirement for increased activities of 5-lipoxygenase in dengue infection. Instead, our data are consistent with an effect on ‘antigen sensing’ although the exact mechanism for this sensing remains to be determined. It remains plausible that contact and exposure to dengue antigens might trigger the release of LTB_4_ from neutrophils, either by directly stimulating the 5-lipoxygenase enzyme or, indirectly, by altering the biochemical milieu and signalling pathways of human neutrophils to enhance antigen recognition [24].

It is not known whether the observed elevation in LTB_4_ is beneficial or deleterious in dengue infection. Previous studies suggested that LTB_4_ may be beneficial in viral-related events. Although LTB_4_ is known primarily for its chemotactic affinity towards leukocytes, LTB_4_ is also capable of activating several functional host defence through secretion of lysosomal enzymes, augmentation of NADPH oxidase activity, nitric oxide and defensins production, and phagocytosis [6,7]. More recent studies indicate that LTB_4_ may be an important mediator involved in the control of viral infection by releasing powerful antimicrobial peptides from peripheral neutrophils [10,25-27]. Over-expression of LTB_4_ may also modulate innate immunity responses by activating effector T cells, which in turn limits the propagation of these viruses [28,29]. On the other hand, elevated LTB_4_ may induce a range of cellular and molecular events that coordinate and amplify inflammatory responses [30], especially within the vasculature [31]. LTB_4_ has been shown to strongly induce monocyte chemoattractant protein-1 (MCP-1) production in human monocytes via activation of ERK1/2 or JNK MAPK [32]. LTB_4_ receptor blockade effectively reduced monocytic foam cell formation and delayed the lesion progression in ApoE knockout mice [33].

Limited data are available on the involvement of monocytes and macrophages in response to 5-lipoxygenase derived metabolites during dengue infection. Elevated levels of hsCRP indicate acute inflammation during the febrile stage, while normalization of their values during defervescence and convalescence suggests diminution of these inflammatory responses following recovery. There is evidence that neutrophil-derived LTB_4_ is involved in inflammatory diseases such as rheumatoid arthritis [34] and atherosclerosis [31]. Significant correlations between circulating hsCRP and LTB_4_ concentrations during DENV-2 infection suggest that LTB_4_ may have important roles in inflammatory events during DENV-2 infection, and this may explain the extent of vascular damage that occurs during DENV-2 infection.

Although dengue infection is characterized by an adult predominance and DENV serotype-2 is the most common circulating strain in Singapore, results from this study may not be applicable to younger patients with dengue serotypes other than serotype-2 and different dengue severities [2]. Our observation also did not exclude the possible contribution of increased levels or activities of 5-lipoxygenase activating protein, coactosin-like protein or LTA_4_ hydrolase induced by DENV antigens in neutrophils.

## Conclusions

These findings add to the relatively sparse human data on the relevance of 5-lipoxygenase-related mediators to human diseases. Coupled with findings from *in vitro* studies that showed augmented LTB_4_ productions from neutrophils following exposure to DENV-2, our data support the ability of DENV to trigger the release of LTB_4_ from neutrophils. These findings call for further studies to assess the importance of the 5-lipoxygenase in modulating viral and host defence factors in dengue infection.

## Methods

### Chemicals

Bovine serum albumin (BSA), calcium chloride, potassium chloride, calcium ionophore A23187, Hepes, MK886, sodium phosphate dibasic, sodium bromide, sodium chloride, sodium hydrogen carbonate, glucose, sodium phosphate monobasic, dextran 500, protease inhibitor P8340, Radio Immunoprecipitation Assay buffer (RIPA), β-mercaptoethanol, glycerol, Tris–HCl, bromophenol blue, glycine, sodium dodecyl sulfate (SDS), isooctane, methyl-cellulose, penicillin, streptomycin, formaldehyde, crystal violet, 2,3,4,5,6-pentafluorobenzylbromide (PFBBr), N,N-diisopropylethylamine (DIPEA) and N,O-Bis(trimethylsilyl)trifluoroacetamide in 1% trimethylsilane (BSTFA + TMS) were purchased from Sigma Aldrich (St Louis, MO, USA); d_8_-arachidonic acid (AA-d_8_) and d_4_-LTB_4_ from Cayman Chemical (Ann Arbor, MI, USA); acetonitrile, magnesium sulphate and sulfuric acid from Merck (Darmstadt, Germany); Ficoll-paque plus® from GE Healthcare (Uppsala, Sweden); phosphate buffered saline (PBS), Hank’s balanced salt solution (HBSS), fetal calf serum (FCS), heat-inactivated fetal calf serum (HIFCS) and RPMI 1640 from Gibco™ Invitrogen (Calsbad, CA, USA); mouse anti-human 5-LOX antibody from BD Biosciences (San Jose, CA, USA); Hybond-C extranitrocellulose membrane and anti-mouse horseradish peroxidase (HRP) from Amersham (Buckinghamshire, UK); Vero cells (ATCC: CCL-81), DENV-2 specific monoclonal antibody 3H5 (ATCC: HB46) and BHK-21 cells (ATCC: CCL-10) from American Type Culture Collection (Manassas, VA, USA); FITC-tagged secondary antibody from Meridian Life Science® Inc (Saco, ME, USA); mouse anti-human glyceraldehyde-3-phosphate dehydrogenase (GAPDH) antibody from Novus Biological (Littleton, CO, USA); and methanol and ethanol from Tedia (Fairfield, OH, USA).

### Study participants

Adult patients, above the age of 21 with acute DENV serotype-2 infection (confirmed using RT-PCR), were included in this study. Two groups of age- and gender-matched healthy and non-dengue febrile subjects served as study controls. Non-dengue febrile controls were selected from patients who presented with acute febrile illness who were tested negative for DENV using RT-PCR and did not manifest a four-fold increase in DENV IgG three months following their acute febrile illness. Clinical information (including age, gender and stage of illness) was obtained and blood samples were collected during the febrile, defervescent and convalescent stages of infection. The study protocol was reviewed and approved by the Domain-Specific Review Board, National Healthcare Group, Singapore, and each patient provided written informed consent prior to study involvement. The demographics of DENV-infected (n = 14, mean age, 44 years; 40% men), healthy (n = 20, mean age, 43; 55% men) and non-dengue controls (n = 15, mean age, 42; 55% men) groups were comparable. The mean duration of symptoms for dengue patients during the febrile stage was 3 days; defervescence, 6 days; and convalescence, 25 days. Similarly, the mean duration of symptoms for non-dengue febrile controls was 3 days.

### Leukotriene B_4_ and arachidonic acid measurements

Blood samples collected in EDTA tubes were centrifuged to obtain plasma, which was stored at −80°C until analysis. Circulating LTB_4_ and AA were determined in plasma using isotope-labelled gas chromatography-mass spectroscopy (GC-MS) as described previously [35,36]. LTB_4_ was quantified using a calibration curve obtained by adding a constant amount of d_4_-LTB_4_ (5 ng) to LTB_4_ (0 to 5000 pg) followed by solvent extraction and derivatization as described previously [35]. Circulating LTB_4_ concentrations were expressed per unit AA.

### High-sensitivity C-reactive protein and phospholipase activities

Blood samples collected in coagulant-free tubes were centrifuged to obtain sera. High-sensitivity C-reactive protein (hsCRP) was measured in serum using Cobas c111 Photometric Analyzer (Roche Diagnostic GmbH, Mannheim, Germany). Commercial assay kits were used to determine plasma phospholipase A_2_ (PLA_2_) activities (Cayman Chemicals, Ann Harbour, MI, USA).

### Peripheral neutrophil isolation

Neutrophils were isolated from neutrophil/erythrocyte pellet from Ficoll-Paque gradient by dextran sedimentation of red cells as described previously [37]. Cell viability was assessed using trypan blue exclusion and was typically >98%.

### Virus preparation

DENV-2 (ST) virus used in this study was a clinical isolate obtained from the Singapore General Hospital, Singapore. These viruses were propagated in Vero cells (ATCC: CCL-81) and harvested when at least 75% of cell monolayer showed cytopathic effects. Confirmation of serotype was performed using immunofluorescence microscopy on the cell monolayer with serotype-specific monoclonal antibody (ATCC: HB46). The culture supernatant was clarified through centrifugation, aliquoted and stored at −80°C until use. The titer of the culture supernatant was determined by plaque assay, where serial 10-fold dilutions of the culture supernatant were added to BHK-21 cells (ATCC: CCL-10) in 24 well plates and incubated for 1 hour at 37°C. The inoculum was aspirated and replaced with 0.8% methyl-cellulose in maintenance medium (RPMI-1640, 2% FCS, 25 mM Hepes, penicillin and streptomycin). After incubation for 5 days at 37°C, the cells were fixed with 20% formaldehyde at room temperature for 20 min. These cells were next treated with 1% crystal violet for 20 minutes. The plaques were counted visually and plaque forming units per mL (pfu/mL) were calculated.

### *In vitro* studies

Freshly isolated neutrophils were resuspended in HBS containing AA (final concentration of 100 μg/ mL) and HBHS [CaCl_2_.2H_2_O (0.18 g/L), glucose (1.0 g/L), Hepes (0.12 g/L), KCl (0.40 g/L), KH_2_PO_4_ (0.06 g/L), MgSO_4_.7H_2_O (0.20 g/L), NaHCO_3_ (0.36 g/L), NaCl (4.00 g/L), Na_2_HPO_4_ (0.04 g/L) and BSA (1.0 g/L) in pure water; pH 7.4] at a concentration of 1 × 10^6^ cells/mL. To assess the effect of viral infectivity on LTB_4_ production, neutrophils were pre-incubated with live and heat-inactivated DENV-2 (10^6^ pfu/mL final concentration). The cell suspension (1 mL) was preincubated with live and heat-inactivated DENV-2 (10^6^ pfu/mL final concentration) at 37°C for 0, 0.5, 2.5, 5 and 7 hours prior to 5-lipoxygenase stimulation. DENV-2 was added using RPMI 1640 as vehicle. The cells were then stimulated with calcium ionophore A23187 (2.5 μg/mL final concentration) at 37°C for 15 minutes. Calcium ionopohore induces neutrophil degranulation and thereby promotes the release of LTB_4_ from neutrophils [38]. The supernatant from cell suspension was collected and stored at −80°C before LTB_4_ extraction and analysis. The cell pellet was resuspended in HBSS and stored at −80°C before 5-lipoxygenase protein measurement. Untreated cells with RPMI 1640 only were used as positive controls while untreated cells incubated with the leukotriene biosynthesis inhibitor MK886 (300 nM) [39] served as negative controls. The release of LTB_4_ from stimulated neutrophils was measured by high performance liquid chromatography coupled with ultra-violet detection [37].

The ability of DENV-2 to infect neutrophils was examined by indirect immunofluorescence assay using DENV serotype 2-specific antibody (ATCC:HB46) and FITC-tagged secondary antibody. The neutrophil suspension (1 mL in HBHS) was preincubated with live DENV-2 (10^6^ pfu/ mL final concentration) at 37°C for 48 and 72 hours prior to infectivity assay. Cell viability was confirmed as at least 80% at the end of incubation using trypan blue exclusion.

A separate experiment was conducted to investigate whether anti-DENV-2 antibody would affect the production of LTB_4_ in human neutrophils. Anti-DENV-2 antibody (ATCC:HB46) was incubated for 5 hours with neutrophils treated with live and heat-inactivated DENV-2. At the end of incubation, the cells were stimulated with calcium ionophore and LTB_4_ production was measured as described above. The amount of LTB_4_ production in neutrophils that were incubated with and without anti-DENV-2 antibodies was subsequently compared.

### Western blot analysis

5-Lipoxygenase expression was determined in freshly isolated neutrophils at 0, 2.5 and 7 hours after incubation with DENV using electrophoresis on SDS gel and subsequent Western blot analysis. Proteins were isolated by suspending neutrophils in RIPA buffer containing protease inhibitor P8340 (20 μL/ mL of RIPA-buffer) for 30 minutes on ice. Protein sample (20 μL) was mixed with 5X sample buffer (10% SDS, 10 mM β-mercaptoethanol, 20% glycerol, 0.2 M Tris–HCl pH 6.8, 0.05% bromophenol blue) and allowed to denature in boiling water for 5 minutes. After centrifugation at 10,000 rpm at room temperature, the supernatant was loaded on 10% SDS-gel. Gel electrophoresis was run at a constant voltage of 120 V, for about 2 hours, or until bromophenol blue front reached the bottom of the gel. The proteins were electrophoretically transferred from the gel onto Hybond-C Extranitrocellulose membrane in a transfer buffer containing 25 mM of Tris–HCl, 192 mM of glycine and 20% methanol at a transfer voltage of 100 V over 90 minutes. The membrane was then cut, based on the size of proteins of interest, to separate the 5-lipoxygenase (79 kDa) and the control protein GAPDH (39 kDa). They were blocked separately overnight in TBS-T buffer (Tris-buffered saline with 1% Tween-20), containing 5% milk, at 4°C or in cold room, with gentle shaking. The blocked membranes were incubated in fresh TBS-T buffer, containing 5% milk and mouse anti-human 5-lipoxygenase (1:1000 vol/vol) or mouse anti-human GAPDH (1:5000 vol/vol) for 1 hour at room temperature. After incubation, the membranes were washed 4 times with TBS-T buffer, before they were treated with anti-mouse HRP secondary antibody (1:5000 vol/vol) for 1 hour at room temperature. After washing with TBS-T buffer, the membranes were realigned on the film cassette, and 5-lipoxygenase and GAPDH proteins were visualized with an ECL Western blot analysis system (Amersham, UK) and exposed to radiofilm (Amersham, UK). The intensity of the protein band on exposed film was measured using ImageJ software (NIH, USA). 5-lipoxygenase protein expression was expressed as the ratio of 5-lipoxygenase protein band intensity to those of GAPDH within the same membrane.

### Statistical analysis

Statistical analyses were performed using SPSS version 15.0 (SPSS Inc, Chicago, Ill). Shapiro-Wilk tests were performed to assess normality of data. Mean ± standard deviation was used to describe normally-distributed data while median (interquartile range) was used to describe non-parametric data. For normally-distributed data, between-group (dengue, healthy control and non-dengue febrile controls) and within-group (febrile, defervescence and convalescence) differences were analyzed using one-way ANOVA analyses with Bonferroni adjustment. For non-parametric data, between-group and within-group differences were analyzed using Kruskal-Wallis analyses with Dunn’s adjustment. Spearman correlation analyses were performed to examine the associations between plasma LTB_4_ and serum hsCRP levels in dengue patients and controls. Between group differences in *in vitro* studies were analysed using ANOVA with Bonferroni adjustment for multiple comparisons. Statistical significance was set at *p* < 0.05.

## Abbreviations

LTB4: Leukotriene B_4_; 5LOX: 5-lipoxygenase; AA: Arachidonic acid; hsCRP: High-sensitivity C-reactive protein; DENV: Dengue virus; DENV-2: Dengue virus serotype-2; PLA2: Phospholipase A_2_; GAPDH: Glyceraldehyde-3-phosphate dehydrogenase; BSA: Bovine serum albumin; RIPA: Radio immunoprecipitation assay buffer; SDS: Sodium dodecyl sulphate; PFBBr: 2,3,4,5,6-pentafluorobenzylbromide; DIPEA: N,N-diisopropylethylamine; BSTFA + TMS: N,O-Bis(trimethylsilyl)trifluoroacetamide in 1% trimethylsilane; PBS: Phosphate buffered saline; HBSS: Hank’s balanced salt solution; FCS: Fetal calf serum; HIFCS: Heat-inactivated fetal calf serum; HRP: Horseradish peroxidase; GC-MS: Gas chromatography-mass spectrometry.

## Competing interests

The authors declare that they have no competing interests.

## Authors’ contributions

RCSS and WML conceived the idea of this work. RCSS, ECHL and AMLQ recruited the dengue patients and collected the samples. WML, AYC, KLMS and CYJL performed the experiments. RSCS and WML performed literature review and statistical analyses. RSCS, BH and EEO critically reviewed and finalized the manuscript. All authors read and approved the final manuscript.
